# Delayed Manifestation of Massive Bilateral Sub-acute Subdural Hemorrhage

**DOI:** 10.7759/cureus.59098

**Published:** 2024-04-26

**Authors:** Anmol K Nagpal, Dr Aditya Pundkar, Charuta Gadkari, Aniket Patel

**Affiliations:** 1 Emergency Medicine, Jawaharlal Nehru Medical College, Datta Meghe Institute of Higher Education and Research, Wardha, IND; 2 Orthopedics, Jawaharlal Nehru Medical College, Datta Meghe Institute of Higher Education and Research, Wardha, IND

**Keywords:** case report, surgical management, mri, minor head trauma, elderly, sub-acute subdural hematoma

## Abstract

Sub-acute subdural hematoma (SASDH) in the elderly is a challenging diagnosis given its insidious onset and nonspecific presentation, particularly following minor head trauma. This case report highlights the clinical features, diagnostic challenges, and management of SASDH in an elderly patient.

A 72-year-old male presented with a five-day history of giddiness, headache, and balance issues, which began suddenly without a significant triggering event. His medical history was notable only for a minor fall approximately one month before presentation, after which he experienced no immediate or significant symptoms. An MRI at an outside hospital revealed bilateral frontoparietotemporal SASDHs with diffuse cerebral edema. The patient underwent a bilateral mini craniotomy for hematoma evacuation and was managed postoperatively with anti-seizure medications and supportive care, resulting in a satisfactory outcome.

The diagnosis of SASDH requires a high index of suspicion, especially in the elderly, who may present with vague and progressive symptoms following minor head trauma. Early and accurate diagnosis via imaging, particularly MRI, is crucial for effective management. Surgical intervention, typically involving hematoma evacuation, significantly improves outcomes in patients with SASDH, underscoring the importance of timely surgical referral and treatment. Elderly patients presenting with unexplained neurological symptoms following even minor trauma should be evaluated for SASDH. Early recognition and intervention are crucial to prevent long-term morbidity and mortality in this vulnerable population.

## Introduction

Subdural hematoma (SDH) is an accumulation of blood between the dura mater and the arachnoid layer of the brain, often categorized into acute, sub-acute, and chronic stages based on the timing of symptom onset post-injury. Sub-acute subdural hematoma (SASDH) typically manifests within 3 to 21 days after head trauma [1]. The clinical presentation can vary significantly, making timely diagnosis challenging, particularly in elderly patients who may present with non-specific symptoms such as confusion, gait instability, or subtle changes in mental status [2].

The incidence of SDH is notably higher in the elderly population, a demographic trend that is partly attributed to increased vascular fragility and brain atrophy. These physiological changes predispose older adults to SDH, even following minor head impacts [3]. Additionally, anticoagulation therapy, common in this age group for various cardiovascular conditions, further increases the risk of developing an SDH after head trauma [4].

Despite its frequency, the delayed presentation of massive bilateral SASDH, as observed in elderly individuals without a history of significant trauma or coagulopathy, is relatively rare and not well-documented in literature. Recognizing such cases is critical, as delayed or missed diagnosis can lead to significant morbidity and mortality. The treatment typically involves surgical intervention, which, in the case of bilateral massive SASDH, may include procedures such as bilateral craniotomy for hematoma evacuation [5]. This case highlights the complexities associated with diagnosing and managing SASDHs in the elderly and reinforces the need for high clinical suspicion following even minor trauma.

## Case presentation

A 72-year-old male was brought to the hospital with complaints of giddiness, headache, and balance issues, which had progressively worsened over five days. His medical history was notable only for a minor fall at home about a month prior, after which he experienced no immediate or significant symptoms. On initial assessment, his airway was patent, breathing stable with an oxygen saturation of 98% on room air, and circulation was normal with a blood pressure of 140/90 mmHg and a pulse rate of 60/min. Neurologically, he was alert and fully oriented, scoring 15/15 on the Glasgow Coma Scale, with equal and reactive pupils and no evidence of focal neurological deficits.

**Figure 1:**
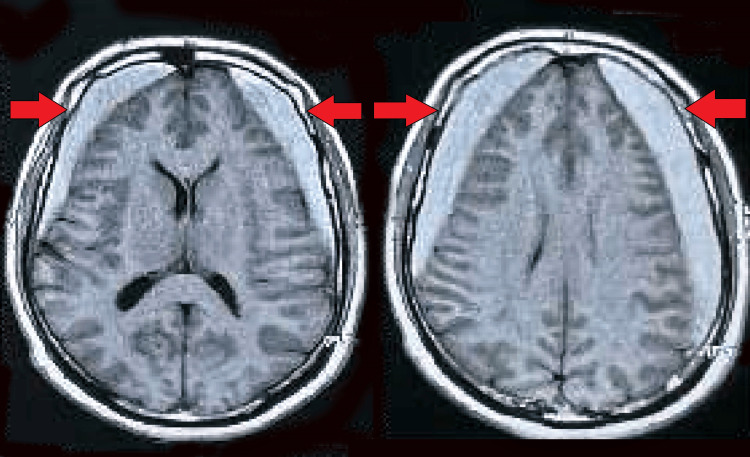
Shows bilateral frontoparietotemporal SASDHs with diffuse cerebral edema SASDH: Sub-acute subdural hematoma

The secondary survey noted that the patient’s symptoms started suddenly five days prior without any obvious triggers. The headache was generalized, not relieved by oral medications, and was non-positional. Balance disturbances were described as a progressive inability to maintain stability while standing or walking, but there were no other associated symptoms like speech disturbances, altered sensorium, or gastrointestinal symptoms. An initial MRI conducted at an outside hospital revealed bilateral frontoparietotemporal SASDHs with diffuse cerebral edema, prompting his transfer to our facility for specialized care (Figure 1).

Upon arrival, laboratory investigations, including a complete blood count, kidney and liver function tests, and a coagulation profile, were all within normal limits. Detailed neurological exams confirmed intact higher cognitive functions, normal speech, and language capabilities, and normal cranial nerve examination. Motor examination revealed normal muscle tone and power in all limbs, but the patient demonstrated a forward fall when standing unsupported. Sensory examination and cerebellar function tests were unremarkable. Cardiovascular and respiratory examinations were also normal.

**Figure 2:**
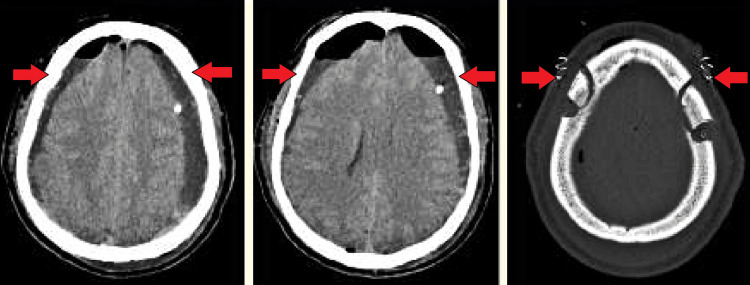
Shows postoperative image of satisfactory resolution of the hematomas

Given the findings and the patient’s progressive neurological symptoms, immediate management included intravenous administration of ondansetron for nausea, levetiracetam for seizure prophylaxis, and paracetamol for pain relief. The patient was then transferred to the neurosurgery ICU, where he underwent a bilateral mini craniotomy for evacuation of the SDHs under general anesthesia the following day. Postoperative recovery was smooth, and follow-up CT scans showed satisfactory resolution of the hematomas (Figure 2).

The patient’s postoperative period was uneventful, and he was discharged to a rehabilitation facility to continue recovery. This case highlights the importance of considering SASDH in elderly patients presenting with nonspecific neurological symptoms following even minor trauma, emphasizing early recognition and intervention to ensure favorable outcomes.

## Discussion

This case exemplifies the critical nature of recognizing SASDH in the elderly following seemingly trivial head trauma. SDHs, particularly in older adults, can present with varied and often subtle clinical features due to cerebral atrophy, which increases the subdural space, making the elderly population more susceptible to SDH even after minor injuries [6]. The delayed onset of symptoms, as observed in our patient, who presented with headaches and balance difficulties several weeks after a fall, is characteristic of SASDH. The pathophysiology underlying SASDH involves venous bleeding, typically from bridging veins between the brain's cortical surface and the dural sinuses. The brain atrophy in elderly patients increases the tension in these veins, predisposing them to rupture from even minor head impacts [7]. In this case, the patient’s initial lack of significant post-fall symptoms and the subsequent development of giddiness and instability indicate the subdural collection gradually reaching a size sufficient to affect brain function.

Importantly, the diagnostic approach must be timely and appropriate. MRI remains a sensitive modality for detecting SDH, capable of identifying the collections and any associated cerebral edema, as was crucial in our patient's timely diagnosis and management [8]. The clinical challenge lies in the nonspecific nature of early symptoms, which may be misattributed to other common conditions in elderly individuals, such as vestibular disorders or stroke. Treatment strategies for SASDH involve addressing the acute symptoms and surgical intervention to evacuate the hematoma. Our approach, involving bilateral mini craniotomy, is supported by literature indicating that surgical management of SDH can significantly improve outcomes, especially in cases where a mass effect or significant midline shift is noted [9]. Furthermore, the use of antiepileptics for seizure prophylaxis in the acute management of SDH, as in our case, is consistent with current recommendations given the high risk of seizures associated with cortical irritation by the hematoma [10].

## Conclusions

In conclusion, this case highlights the clinical subtleties and complexities associated with diagnosing and managing SASDH in the elderly. Our patient's presentation underscores the importance of considering SASDH in elderly patients who present with nonspecific neurological symptoms following even minor head trauma. Early diagnostic imaging, timely surgical intervention, and appropriate postoperative care are crucial for favorable outcomes in such cases. This case also serves as a reminder of the potential for delayed symptomatology in SDHs, emphasizing the need for healthcare professionals to maintain a high suspicion index and act promptly, even when initial symptoms appear mild or unrelated. Prompt recognition and intervention can significantly improve the prognosis in elderly patients suffering from this potentially life-threatening condition.
